# Modeling intraindividual variability in affect (MIVA): Formalized theoretical approach, computational model, and parameter recovery study

**DOI:** 10.3758/s13428-026-03122-w

**Published:** 2026-07-20

**Authors:** Maria Wirth, Andreas Voss, Stefan T. Radev, Klaus Rothermund

**Affiliations:** 1 Department of Psychology, Friedrich Schiller University Jena, Am Steiger 3/1, 07743 Jena, Germany; 2 Department of Psychology, Heidelberg University, Heidelberg, Germany; 3 Department of Cognitive Science and Center for Modeling, Simulation, and Imaging in Medicine, Rensselaer Polytechnic Institute, Troy, NY USA

**Keywords:** Affect dynamics, Simulation-based inference, Emotional reactivity, Emotion regulation, Mathematical modeling, Neuronal networks

## Abstract

No matter how angry, sad, or happy we are, eventually, we will feel different. Studying this ebb and flow of affective experience in daily life provides important insights into psychological functioning and well-being. We have developed a parsimonious formalized model of intraindividual variability in affect (MIVA), resting on the assumption that such affective changes reflect transactions between an individual and their proximal environment. We provide an outline of its theoretical background, scope, and mathematical formulation. We situate MIVA within the research field of affect dynamics and illustrate the models’ behavior under realistic conditions using a simulation study. We use simulation-based inference to train a custom neural network on MIVA simulations, which we employ to rapidly estimate the model’s parameters on a multitude of synthetic experiments with different configurations. Our simulation study demonstrates that the synthesis between a computational model and probabilistic neural networks results in an efficient and flexible tool for model-based inference of affect dynamics. Our simulation study also offers insights into the data requirements for a precise recovery of the model’s parameters and recommendations for future data collection. The potential of MIVA for providing insights into affect dynamics is discussed.

Affective experience is inherently dynamic. Within a day, an individual may experience all possible shades of feelings, from the ecstasy of joy to the depths of despair. Describing the principles and regularities underlying affective fluctuations, identifying the processes driving them, and understanding the implications they have for psychological functioning are the aims of affect dynamic research^1^ (Koval & Kuppens, 2024; Kuppens, 2015; Kuppens et al., 2010). Past affect dynamic work has relied on two approaches for extracting relevant information concerning affect dynamics: simple summary statistics (such as the intraindividual standard deviation or first-order autocorrelation) and model-based approaches (Dejonckheere et al., 2019; Loossens et al., 2020). Model-based approaches try to capture the observed variability in a process of interest by fitting a computational model to empirical data (Ram et al., 2014). Inspired by contemporary affect dynamic models (Chow et al., 2005; Kuppens et al., 2010; Loossens et al., 2020), we developed the Model of Intraindividual Variability in Affect (MIVA; Wirth et al., 2022, 2023) with a specific focus on formalizing the relation between fluctuations in hedonic valence and individual differences in responding to affect-eliciting events, an issue that is still of high relevance in affect dynamic research (Ryan et al., 2025).

Although simple summary statistics have been criticized as descriptive and offering little information concerning underlying processes (Adolf et al., 2017; Deboeck et al., 2009; Jahng et al., 2008; Mestdagh et al., 2018; Trull et al., 2015), they still seem to dominate empirical research on affect dynamics (Vanhasbroeck et al., 2022). As model-based approaches, including our own, might be difficult to understand, implement, and communicate, in this article, we seek to achieve two goals: First, we elaborate on MIVA’s theoretical background, its formalization, and embed MIVA within the research field of affect dynamics. Second, capitalizing on the amortized simulation-based inference (Cranmer et al., 2020; Radev et al., 2022), we introduce a new, efficient inference method for estimating MIVA model parameters. Using the results of a comprehensive simulation study, we provide specific recommendations for data collections aiming to apply the MIVA model (i.e., number of days and measurements per day). To conclude, we will discuss the potential of MIVA for providing insights into affect dynamics.

## MIVA: Basic concepts and their relations

We developed MIVA to integrate the translational process between external events and affective experience into a model-based approach, as this had been pointed out in previous work as an important step for better understanding affect dynamics (Adolf et al., 2017; Chow et al., 2005; Kuppens et al., 2010; Vanhasbroeck et al., 2021). Specifically, the MIVA model intends to provide a useful heuristic for characterizing fluctuations in hedonic valence and tries to capture interindividual differences in response to affective-eliciting events.

We will shortly outline the model’s parameters and their relations; interested readers can find more details in the Supplemental Material (https://osf.io/spxf8/files/2u35k). Generally, the model assumes that fluctuations in affective experience reflect transactions between an organism and its proximal environment (Frijda, 2007; Larsen, 2000; Reich et al., 2003). Rather than objective characteristics, changes in affect are based on an individual’s subjective appraisal of a situational change or event (Frijda, 1993; Roseman, 1996; Scherer, 2009). Following many appraisal theories (Frijda, 1993; Scherer, 1993; Smith & Ellsworth, 1985), the model includes a parameter for hedonic event quality, known as event pleasantness. If an event is appraised as pleasant, the elicited affect is positive. If the event is appraised as unpleasant, the resulting affect is negative.

How events and their appraisal can result in changes in affect across shorter and longer time scales is captured by two model parameters: *Affective reactivity* encompasses individual differences in the extremity of short-term changes in affective experience caused by situational changes (Bolger & Zuckerman, 1995; Davidson, 1998; Mroczek & Almeida, 2004; Rosenberg, 1998). Individuals with high affective reactivity show substantial alterations in affective experience if they encounter situational changes, compared to their average affect level. Individuals with low reactivity only show minor changes in affect. The impact of valent events on affective experience thus depends on how a person generally reacts to events and on how the person appraises specific events. Given that affective reactivity is usually higher for events that are appraised as unpleasant compared to those appraised as pleasant (Backs et al., 2005; Bylsma et al., 2011), our model differentiates between affective reactivity for pleasant and unpleasant events.

*Affect regulation* captures interindividual differences in longer-term changes in affective experience that originated from affect-eliciting events. Specifically, it encompasses any process that aims at changing the intensity, quality, or duration of affective experiences and/or expressions (Gross, 2013; Koole & Rothermund, 2011). Individuals with high affect regulation show a steep and fast decline of affective reactions. For individuals with low affect regulation, affect persists over longer periods. How fast the impact of events can be regulated also depends on the event's pleasantness. Affective reactions to unpleasant events are attenuated faster and more strongly than affective reactions to pleasant events (Hemenover, 2003; Volokhov & Demaree, 2010). Thus, our model also differentiates between affect regulation of pleasant and unpleasant event impact (for similar reasoning, see Ong et al., 2025).

Additionally, our model accounts for interindividual differences in an individual’s typical affective experience in the absence of affect-eliciting events, called *anchoring*. This state may be predominantly positive or negative rather than neutral (Diener & Diener, 1996). Anchoring exhibits cross-situational stability and is an independent source of affective experience (Alessandri et al., 2014).

Taken together, we have identified the basic processes (i.e., event appraisal, reactivity, regulation, accumulation, and anchoring) underlying the variability and fluctuations in everyday affective experiences and have developed a conceptual model based on the central insights that have emerged from the research on affect generation and fluctuation.

## Mathematical formalization of MIVA

So far, the presentation of MIVA has been restricted to a verbal account of its most important principles. To make the model accessible to empirical research and to allow rigorous testing of predictions derived from it, its principles were translated into a mathematical model (for an overview on formalizing verbal affect dynamic theories, see, e.g., Haslbeck et al., 2022).

MIVA focuses on explaining variability in hedonic valence (positive–negative), which is not necessarily the only dimension of affect but is fundamental to its description (Barrett & Russell, 1999; Kuppens et al., 2013; Russell, 2003; Schiller et al., 2024; Watson et al., 1999). Although the question of whether valence is best conceptualized as one bipolar dimension or as two separate unipolar dimensions is debated (Green et al., 1993; Kuppens et al., 2013; Tellegen et al., 1999), we assume that affect is assessed on a bipolar scale. Truly unipolar scales (not interpreted as bipolar by participants) require a two-step procedure: first, asking whether individuals felt happy/unhappy, and then asking to what extent they felt happy/unhappy (Schmukle & Egloff, 2009; Segura & González-Romá, 2003). Affective experience is rarely assessed using this two-step procedure, and many affect dynamic researchers often use single-item measures (Dejonckheere et al., 2022; Fritz et al., 2024; Laurenceau et al., 2025), which are an efficient, sufficient, and low-burden assessment tool for momentary affect (Cloos et al., 2023; Tuerlinckx et al., 2026). Moreover, a unidimensional bipolar approach as employed in the MIVA model is already sufficient to explain a wealth of affect dynamics (cf. Zavlis et al., 2025). Conceptualizing hedonic valence as two separate dimensions would severely restrict MIVA’s empirical applicability. Moreover, many studies show rather low co-occurrence of positive and negative affect (Riediger et al., 2014; Schmukle & Egloff, 2009; Schneider & Stone, 2015; Watson & Stanton, 2017). Similarly, event pleasantness is conceptualized as being assessed on a bipolar scale.

The following principles were considered during the formalization process: (1) The mathematical formulation needs to allow a nonlinear mapping between event pleasantness and affective experience (for similar reasoning, see Zavlis et al., 2025), so that differences in event pleasantness result in larger differences in affect when event pleasantness is low (i.e., close to zero), compared to the same pleasantness difference for extreme events (Thagard & Nerb, 2002; Tong et al., 2009). (2) Affective predictions need to be confined to the scale range of the assessed affective outcome. A sigmoid function can meet these requirements, as it is constrained by a pair of horizontal asymptotes as x →  ± ∞, and it is often used when a specific mathematical formulation is lacking (Gibbs & Mackay, 2000). To take advantage of the distributional properties of the sigmoid function (convex for values less than 0 and concave for values greater than 0), affect and event pleasantness need to be assessed on a bipolar scale (with unpleasant affect and events being indicated by values smaller than 0).^2^ This also helps to communicate the bipolarity of the affect measure to participants (Schimmack, 2003).

The previously described ideas and principles are expressed in the following equation:

1.1 $${A}_{j}=\frac{r}{1+ {e}^{-(a+Ru\, \bullet \,{ Iu}_{j}+Rp\, \bullet\, { Ip}_{j})}}- \frac{r}{2}$$

where *A*_*j*_ is the predicted affective experience at an observation *j*, measured on a bipolar scale (negative–positive affect) with range *r* and centered around zero*.*^3^* a* is the parameter for anchoring, which represents the mean affect level when no valent event is experienced. *Ru* and *Rp* are the reactivity parameters for unpleasant and pleasant events, respectively. *Iu*_j_ and *Ip*_j_ provide accumulated event pleasantness (*p*) from all current and past unpleasant and pleasant events, respectively, and are computed as follows:

1.2 $$I{p}_{j}={\sum}_{{E}_{p, t<{t}_{j}}}{P}_{{E}_{p}}\cdot \frac{1}{1+Dp\cdot {\Delta \mathrm{t}}_{E}}$$

1.3 $$I{u}_{j}={\sum}_{{E}_{u, t<{t}_{j}}}{P}_{{E}_{u}}\cdot \frac{1}{1+Du\cdot {\Delta \mathrm{t}}_{E}}$$

In Eqs. 1.2 and 1.3, ${E}_{p}$ and ${E}_{u}$ are pleasant and unpleasant affect-eliciting events, respectively, occurring before time ${t}_{j}$ with a specific pleasantness ${P}_{E}$, and $\Delta {t}_{E}$ is the time that has passed since the event (${\Delta}_{t}={t}_{j}-{t}_{E}$). *Dp*_*i*_ and *Du*_*i*_ represent the magnitude of regulation that dampens the impact of pleasant or unpleasant events, respectively. For a current event (${\Delta}_{t}=0$), the discounting function equals 1, meaning that a current event exerts its strongest influence on affect. Past events are discounted and have less impact on current affect. This discounting is relatively rapid shortly after the occurrence of an event but falls more slowly thereafter (Setodji et al., 2019; Verduyn et al., 2009, 2012). *Ip*_j_ is always positive (because ${P}_{{E}_{p}}$ is positive) and leads to an increase in affect, *Iu*_j_ is always negative (because ${P}_{{E}_{u}}$ is negative) and leads to a decrease in affect.

**Fig. 1:**
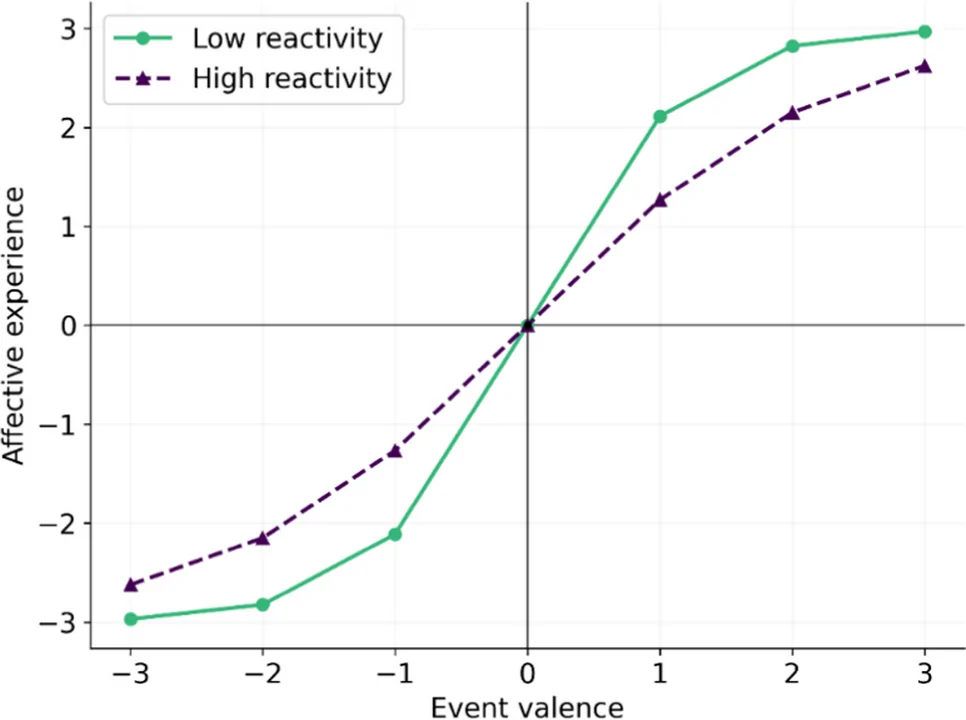
Affective experience as a function of event valence and interindividual differences in reactivity

The behavior of MIVA is similar to previous models of affect dynamics (Chow et al., 2005; Kuppens et al., 2010). Once a valent event occurs and is appraised as such, affect (*A*_*j*_) will show a deviation from the average affect level (*a*). The direction of the fluctuation depends on event pleasantness (*P*_*E*_), with pleasant events increasing affect such that it becomes more positive or less negative. Unpleasant events, in turn, decrease affective experience such that it becomes more negative or less positive. The amplitude of the fluctuation depends on the reactivity to different situational changes (*Ru* or *Rp*). The effect of interindividual differences in reactivity on the relation between event pleasantness and resulting affective experience can be seen in Fig. 1. To reach the same level of negative affect, an individual with high reactivity (dashed line) needs to experience a moderately unpleasant event, while someone with low reactivity (solid line) needs to experience a highly unpleasant event.

**Fig. 2:**
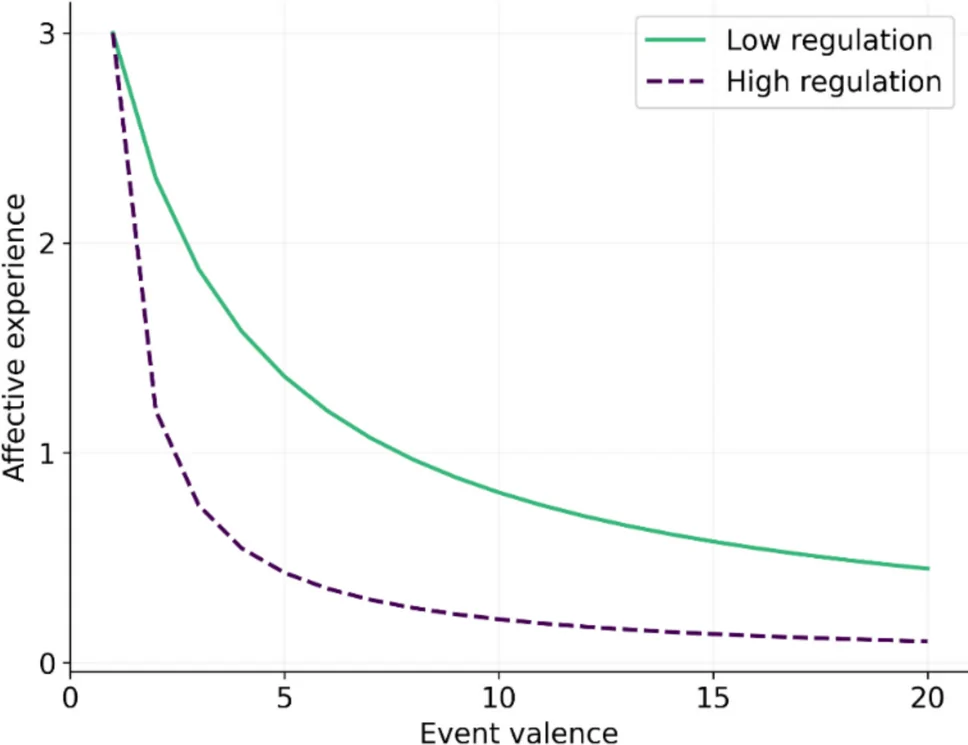
Changes in affective experience as a function of time since the event (in minutes) and interindividual differences in regulation

If no other affect-eliciting events are experienced, affective experience will return to its average level depending on the strength of the regulatory processes (*Du* or *Dp*). The effect of interindividual differences in affect regulation on changes in affective experiences after an affect-eliciting event can be seen in Fig. 2. Individuals with high affect regulation (dotted line) show a fast decline in affective experience across time compared to individuals with low affect regulation (solid line).

**Fig. 3:**
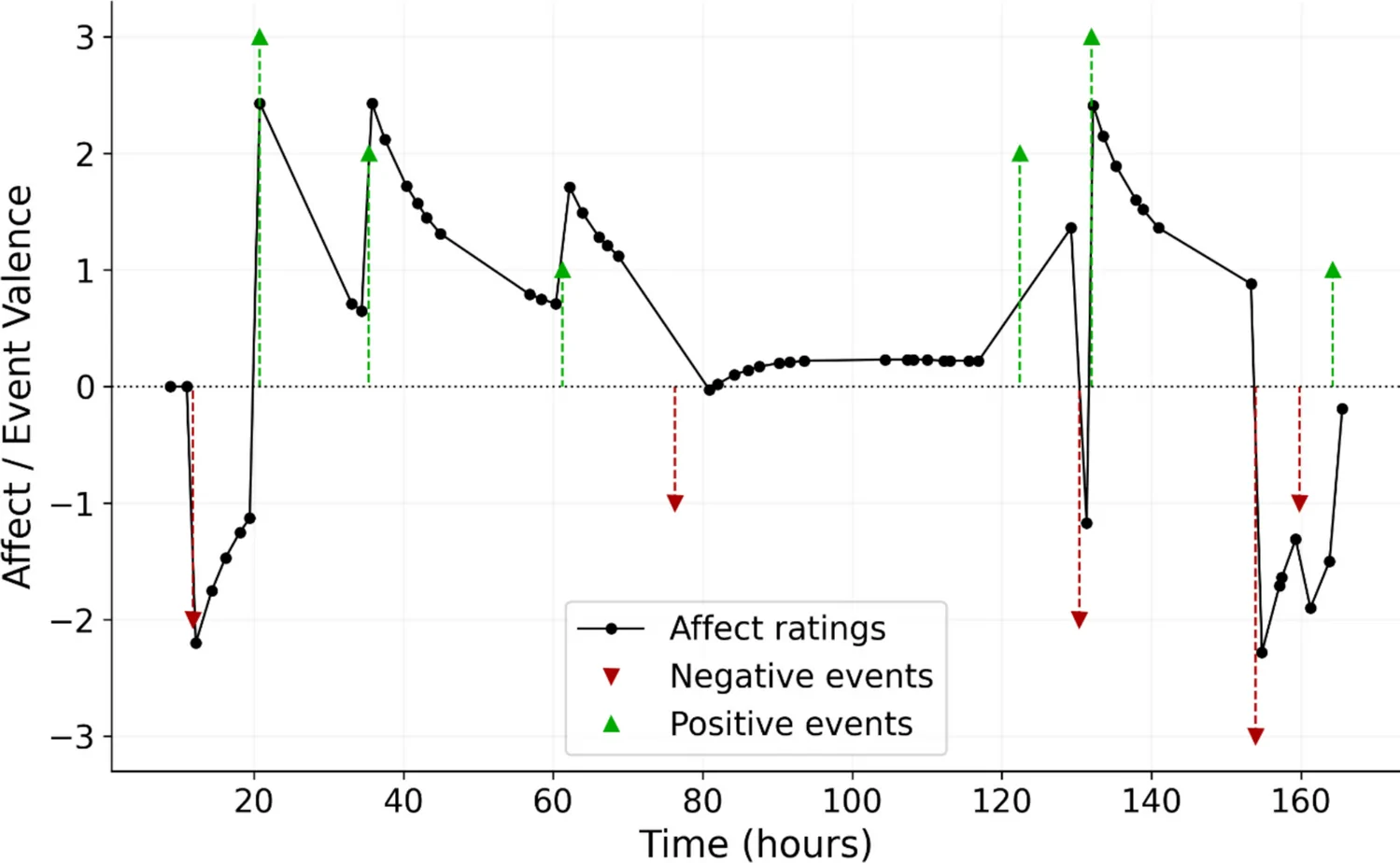
Simulated affective experience for one hypothetical participant. *Note*: Black dots represent the affect ratings, and vertical lines show valent events. Vertical lines above the midpoint indicate positive events, and vertical lines below the midpoint indicate negative events

If other events are experienced, the course of the affective experience depends on the cumulated impact of all past events. An example of the impact of event pleasantness on affect dynamics can be seen in Fig. 3, which shows a simulated dataset for one hypothetical participant, reporting affective experience, event occurrence, and pleasantness for 7 days and eight measurements per day. Affect and event ratings range between − 3 (highly unpleasant/negative) to 3 (highly pleasant/positive). An average number of two events per day (following a Poisson distribution) and the following MIVA parameter values were used for the simulation: *a* = 0.0, *Ru* = 1.0, *Rp* = 1. 0, *Du* = 0.2, *Dp* = 0.2. Events occurred slightly before the corresponding measurement occasion. It can be seen that affect-eliciting events (vertical lines) have a strong initial impact on affective experience (black circles) that gradually declines thereafter. The first unpleasant event reported on the third measurement occasion decreased affect (it became more negative). On subsequent measurement occasions, the impact of this event is discounted, and affect becomes less negative. The occurrence of the first pleasant event at the eighth measurement occasion increased affective experience (it became more positive). On subsequent occasions, the impact of this pleasant event is discounted, and affective experience becomes less positive.

## Computational models of affect dynamics

Before we turn to our simulation study, we situate the MIVA model within the landscape of computational models in affect dynamics. Computational models in affect dynamics are statistical or mathematical models that formalize how one believes the target system (i.e., affect) works (cf. Vanhasbroeck et al., 2021), which has several advantages over verbal accounts of affect dynamics (Haslbeck et al., 2022; Ryan et al., 2025; Vanhasbroeck et al., 2021). Computational models allow us to quantify how complex affect processes evolve dynamically over time within individuals and how this differs across individuals (Lafit et al., 2022; Tuerlinckx et al., 2026; Vanhasbroeck et al., 2021). While many different computational models of affect dynamics exist (Ryan et al., 2025; Tuerlinckx et al., 2026; Vanhasbroeck et al., 2021), it has been reasoned that many researchers desire models that are sensitive to the serial dependence of the data, can quantify regulatory processes, and can take contextual factors into account (Adolf & Ceulemans, 2025; Kuppens & Verduyn, 2017). Especially, the inclusion of contextual information into computational models should greatly enhance the understanding of why affect fluctuates (Vanhasbroeck et al., 2021).

As affect dynamic researchers can pick from a variety of computational models (Cui et al., 2023; Tuerlinckx et al., 2026; Vanhasbroeck et al., 2021), we want to discuss similarities and differences between MIVA and the most widely used models. The field’s main reference point seems to be (vector) autoregressive models (Tuerlinckx et al., 2026; Vanhasbroeck et al., 2022). They are used to study the serial dependence of the affect system (also known as inertia; Koval & Kuppens, 2012, 2024; Koval et al., 2021) and, in terms of vector autoregressive models, also the cross-lagged dependencies of two or more affect variables. Thus, (vector) autoregressive models can be used to model affect dynamics for unidimensional approaches to affect (such as the MIVA model^4^) but also discrete affect approaches, modeling, for example, the dependence of positive and negative affect. It has been conceptualized and empirically shown (Koval et al., 2015) that inertia can capture regulatory processes.

There are, however, several challenges of applying (vector) autoregressive models to the study of affect dynamics using experience sampling data (Berkhout et al., 2025; Cui et al., 2023; Lafit et al., 2022; Vanhasbroeck et al., 2021). For example, the prerequisite of equally spaced observations, which is usually not met by empirical data, leads to biased estimates across different interval lengths (Berkhout et al., 2025). More importantly, unlike the MIVA model, (vector) autoregressive models do not explicitly model contextual information. Rather, it is assumed that dynamic errors or innovations account for context (Adolf & Ceulemans, 2025).

To circumvent problems of discrete time models, such as the (vector) autoregressive model, continuous time models have been proposed, the most popular ones in affect dynamic research being the Dynamic Affect model (DynAffect; Kuppens et al., 2010) and the Affective Ising Model (AIM; Loossens et al., 2020, 2022). Generally, MIVA, DynAffect, and AIM assume that affective processes unfold continuously across time; however, the facets of affective experience modeled differ across models.

MIVA focuses on affective valence (unidimensional, bipolar), whereas DynAffect models valence and arousal (bidimensional, bipolar), and the AIM focuses on positive and negative affect (bidimensional, unipolar). Accordingly, the data required for parameter estimation varies, with all models requiring an indicator of measurement time as well as one or two affect measures. As MIVA focuses on event-related changes in affect, it also requires the collection of event time and a rating of event pleasantness. This should be kept in mind when designing experience sampling method (ESM) studies or applying the MIVA model to existing datasets.

All models assume that fluctuations occur due to perturbations of the system, but how perturbations—that is, changes in context—are incorporated into the computational model, differs. In the DynAffect model and the AIM, context changes are only modeled by stochastic deflections (for AIM applications with input function linking context changes, see Vanhasbroeck et al., 2022, 2024), overlooking how these changes might shape affective experience (Prince et al., 2025). In contrast, MIVA incorporates context changes in terms of the occurrence of affect-eliciting events and the appraisal thereof explicitly into the mathematical model formulation.

As shown in Table 1, while the estimated parameters of MIVA and the DynAffect model can be interpreted in terms of psychological processes (e.g., regulation), the AIM does not attach strong interpretations to its parameters in terms of psychological processes but focuses on distributional and qualitative features of affective experience (Loossens et al., 2022). In comparison to MIVA and AIM, the DynAffect model is unbounded, thus potentially producing nonsensical predictions that fall outside of the data range (Vanhasbroeck et al., 2022, 2024). Also, the DynAffect model does not capture potential nonlinearities of the affect system, while the MIVA assumes a nonlinear mapping between event pleasantness and affective experience, and the AIM itself is a nonlinear stochastic model. Interestingly, DynAffect and AIM assume that recovery is represented by exponential decay. However, it has been argued that the assumption of an exponential decay is unsubstantiated (Zavlis et al., 2025). The MIVA model, in turn, assumes hyperbolic decay but could, in principle, also handle other decay functions (e.g., linear).

**Table 1:** Comparison between MIVA, DynAffect, and AIM

Model	Scope of model	Estimated parameters	Data needed to estimate parameters	Nonlinearity of affect system	Bounded model	Decay function	Software	Empirical applications	Sample size recommendation
Model of Intraindividual Variability in Affect (MIVA)	Characterize interindividual differences in affective valence dynamics by incorporating event effects	*Anchoring*: a person’s average levels of experience when events are absent *Reactivity*: short-term changes in affective experience related to event *Regulation*: longer-term changes in affective experience following an event	Time, affective experience, event pleasantness, event time	Yes	Yes	Exponential, other options possible (e.g., linear)	RStan; BayesFlow: Python	Affect dynamics and well-being (Wirth et al., 2022) Age differences in affect dynamics (Wirth et al., 2023)	Yes
Dynamic Affect Model (DynAffect)	Characterize interindividual differences in core affect dynamics (valence and arousal)	“*home base*”: a person’s average levels of experience *Variability*: fluctuation in affective experience across time “*Attractor strength*”: stability of affective experience	Time, valence, and arousal experience	No	No	Exponential	RJags; Bayesian Hierarchical Ornstein–Uhlenbeck Modeling toolbox; MATLAB	Relation to (emotional) traits; (Kuppens et al., 2010; Oravecz & Brick, 2019) Age differences (Wood et al., 2018) Psychopathology (Ebner-Priemer et al., 2015)	No
Affective Ising Model (AIM)	Capturing nonlinear phenomena in the dynamics of positive and negative affect	*Λ1, Λ2*: positive feedback parameters *Λ12*: mutual interaction between the positive and negative pools Θ1, Θ2: thresholds; how easy it is to excite positive or negative pool *N*1, *N*2: entropy of the system, that is, which affect states are more probable *D*: diffusion constant, the smaller *D*, the slower the affect state evolves	Time, positive and negative affective experience	Yes	Yes	Exponential	Julia software package	Model comparison (Loossens et al., 2020; Vanhasbroeck et al., 2022, 2024) Relation between discrete affective experiences (Tongco-Rosario et al., 2024)	No

All models have undergone empirical testing; for example, the five-parameter MIVA model was the best-fitting model compared to alternative models in which one or more aspects of the MIVA model were absent (Wirth et al., 2022). While empirical studies of the DynAffect and MIVA parameters have shown external validity (e.g., in relation to well-being indicators), the empirical applications of the AIM are mostly focused on its performance compared to other computational models (see Table 1).

Empirical applications of the computational models hinge on how well researchers understand the model, its parameters, and scope, but it also seems important which software is required for parameter estimation, as well as understanding how many data points are needed to precisely estimate the parameters. DynAffect, as the earliest introduced model, has been implemented using different software and packages; the AIM has capitalized on a single package implemented in one software. While previous applications of MIVA have relied on customized code using the rstan package (Stan Development Team, 2025), our simulation study capitalizes on simulation-based inference (SBI; Cranmer et al., 2020). SBI can reduce computational costs for large-scale parameter recovery studies by training neural networks on simulations. Once trained, these networks can perform inference almost instantaneously and across multiple datasets with varying configurations (e.g., numbers of measurements), a property known as *amortized inference.* Training a generative network for all existing and future data compatible with a model and sharing it with other researchers increases the reproducibility of findings and the estimation efficiency (no further training needed) in terms of new data. Here, we utilize SBI as implemented in BayesFlow 2 (Kühmichel et al., 2026), which allowed us to conduct a comprehensive, otherwise costly simulation study.^5^ This simulation study provides insights into how design factors of ESM studies (i.e., measurement days and occasions per day) are related to a precise estimation of the MIVA parameters. This information is currently not available for DynAffect and AIM. More general advice on the design of ESM studies can be found, for example, in the handbook published by Myin-Germeys and Kuppens (2026).

## Simulation study

The value of a computational model for its research field hinges mostly on its ease of use and the availability of guidance with regard to the empirical data collection in terms of, for instance, sample size. This simulation study had two aims. Firstly, a computational bottleneck of the extensive parameter estimation is addressed by using amortized SBI as implemented in BayesFlow 2 (Kühmichel et al., 2026) for the MIVA model. Secondly, we provide guidance on data collection and on the application of the MIVA model to already collected data. In a comprehensive simulation study in which our MIVA model was assumed to be the true data-generating process, we investigated the estimation precision across varying numbers of observations (i.e., study days and measurements per day).

Amortized SBI is designed to offset the cost of Bayesian inference over multiple models, datasets, and contextual factors (e.g., dataset sizes) by learning approximate posteriors *globally* over the entire range of plausible parameters and datasets (Radev et al., 2022, Fig. 1b).

Compared to Markov Chain Monte Carlo (MCMC)-based estimation using Stan (Carpenter et al., 2017), which we applied for estimating MIVA parameters in previous publications (Wirth et al., 2022, 2023), amortized SBI distills posterior sampling into a neural network that becomes an expert in “inverting the model.”^6^ Following simulation-based training, the network can obtain posterior estimates from any empirical or simulated dataset compatible with the MIVA model at a tiny fraction of the time required for training. This idea has become a cornerstone of modern probabilistic modeling workflows (Bürkner et al., 2023, 2025; Deistler et al., 2025; Lavin et al., 2021; Zammit-Mangion et al., 2023) and can be viewed as a complementary approach to MCMC whenever repeated model (re)-fits are too costly or computationally infeasible (Li et al., 2024).

In BayesFlow, amortized posterior estimation involves two separate neural networks trained jointly. The summary network learns to embed datasets with varying numbers of measurements into vector representations of a fixed size. Specifically, the summary network takes raw data—which in MIVA's case includes affect ratings, event timings, and event pleasantness ratings for each participant—and compresses it into a fixed-length vector of numbers. This is necessary because different participants have different numbers of observations and events, but the inference network needs fixed-size input. The embeddings can be interpreted as learned sufficient summary statistics h(x), which can be used in place of the full dataset x for conditioning the posterior $p\left(\theta \right| x)$, that is, $p\left(\theta \right| h(x))\approx p\left(\theta \right| x)$. Due to joint training, these statistics are guaranteed to be maximally informative in principle (Radev et al., 2022). A sufficient statistic captures all the information in the data relevant to parameter estimation—once you have it, the raw data add nothing more. For example, for estimating the mean of a normal distribution, the sample mean is sufficient. Approximately sufficient means that the summary network learns statistics that capture nearly all relevant information, without requiring us to derive them analytically. The network discovers through training which features of the data are most diagnostic of each parameter.

The inference network is realized as a generative network (e.g., a score-based diffusion model; see Arruda et al., 2025, for more details) that approximates the full joint posterior of model parameters given a dataset embedding. During training, true model parameters are drawn from prior distributions, and synthetic datasets are simulated from MIVA. Thus, the inference network takes those summary statistics and outputs the posterior distribution over the model parameters. It has learned (during training on thousands of simulated datasets) how patterns in the summaries relate to the underlying parameter values.

Then the neural networks are trained via standard backpropagation, so that the networks learn to approximate the true posteriors. Following training, the networks’ weights are “frozen,”^7^ and no further learning is required, amortizing the cost of upcoming evaluations.

### Study design

The majority of studies interested in affect dynamics use data of intensive longitudinal designs, including experience sampling methods (ESM), in which participants are asked about affect and related experiences multiple times per day for several days or weeks (Cloos et al., 2024; Hamaker et al., 2015; Vogelsmeier et al., 2024). In this simulation study, we will cover the most common designs found in ESM studies (Pirla et al., 2022; Riediger & Rauers, 2018; Seizer et al., 2024; Wrzus & Neubauer, 2023; Zawadzki et al., 2019) concerning the length of data collection, with 7 to 21 days and 5 to 20 measurement occasions per day. As data in ESM studies can follow different sampling schemes, we decided to mimic one that is most common, namely, interval-based or time-contingent sampling in which daily measurement occasions are scheduled at prespecified times within a time interval (Cloos et al., 2024; Conner & Mehl, 2015; Tuerlinckx et al., 2026). The exact timing of each measurement occasion per day was determined by using the following common procedure: For each day, a time window of 12 h (between 9 AM and 9 PM) was divided equally by the number of notifications per day, and within the resulting time slots, one measurement was scheduled randomly. Although measurement occasions were sampled at discrete time points, time is treated as continuous when estimating the MIVA parameters.

Determining the number of affect-eliciting events per day was informed by our previous studies (Wirth et al., 2022, 2023), with an average rate of about three events per day. The number of events per participant was drawn from a Poisson distribution, with the added provision that there could be no more than one event between two consecutive measurement occasions. This restricts the maximum number of events to the product of days and measurements per day. Further, we only included datasets that entailed at least two events over the entire simulated data collection, as an estimation of reactivity and regulation is otherwise intractable.^8^ For each simulated dataset, MIVA parameters were drawn from the following prior distributions: For anchoring, a truncated normal prior (N(0, 1); [− 3, 3]) was used; for reactivity and regulation, truncated gamma priors (G(2, 2); [0.1, 3.0]) were adopted. These distributions are based on previous results from the MIVA model (Wirth et al., 2022, 2023).

Event pleasantness ratings were simulated on a seven-point scale, as this is one of the most common response scales (Schimmack, 2003). The scales had a symmetrical response format to indicate a bipolar scale: − 3 indicating highly unpleasant and + 3 indicating highly pleasant events. Pleasantness for each event was sampled randomly from six possible values: − 3, − 2, − 1, 1, 2, 3 (i.e., excluding 0). Affect ratings were simulated on a scale from − 50 to + 50 to allow for the detection of more fine-grained changes.

Given that actual assessments of affective experience are prone to measurement error (Dejonckheere & Mestdagh, 2021), simulated affect ratings (*A*_*ij*_) were assumed to be influenced by random noise. For this purpose, Gaussian noise with standard deviation s was added to the exponent in Eq. (1.1):

2 $${R}_{j}=\frac{r}{1+ {e}^{-(a+Ru\, \bullet\, { Iu}_{j}+Rp \,\bullet \,{ Ip}_{j}+e)}}- \frac{r}{2}, with\, e\sim N\left(O,s\right)$$

where *s* was drawn from a uniform distribution between 0 and 1 for each simulated participant.

### Network architectures and simulation-based training

Since simulations from the MIVA model are extremely fast, we used online training in which the networks never see the same simulation twice. Accordingly, we trained the networks for 1,000 epochs, with 200 mini-batches per epoch and a batch size of 32 for a total of 16,000,000 simulations using the Adam optimizer with an initial learning rate of $1\times {10}^{-4}$. In each batch, we varied the number of days and number of measurements per day between 7 and 21 and between 5 and 20, respectively. The inference network is implemented as a consistency model (Schmitt et al., 2024), which is a few-step neural sampler designed for fast inference. The summary network is implemented as a custom encoder-only transformer designed to reduce time series of varying lengths into fixed-size vectors. Training, validation, and inference were implemented in the BayesFlow Python library (version 2.0.8; Kühmichel et al., 2026). The networks were trained on a single graphics processing unit (GPU) machine equipped with an NVIDIA® A2000 graphics card.

### Performance validation

To evaluate the trained inference model, we systematically varied the study design along two dimensions: the number of days and the number of measurements per day. Specifically, we considered eight levels of study duration (7, 9, 11, 13, 15, 17, 19, 21 days) and four levels of measurements per day (5, 10, 15, 20), resulting in 32 distinct conditions in total. For each design, we generated 500 independent test simulations. For every simulated dataset, the inference network then produced 1,000 posterior samples for the five model parameters: anchoring, positive reactivity, negative reactivity, positive regulation, and negative regulation*.* In total, the analysis comprised 16,000 simulated test cases (32 × 500) and 16,000,000 posterior samples (32 × 500 × 1,000).

Training of the networks took 70 min. Despite the scale of the evaluation, inference was computationally very efficient: generating all posterior samples across all designs and test simulations took less than 3 min in total for 16 million posterior samples (excluding simulation and metric computation).

#### Simulation-based calibration check

**Fig. 4:**
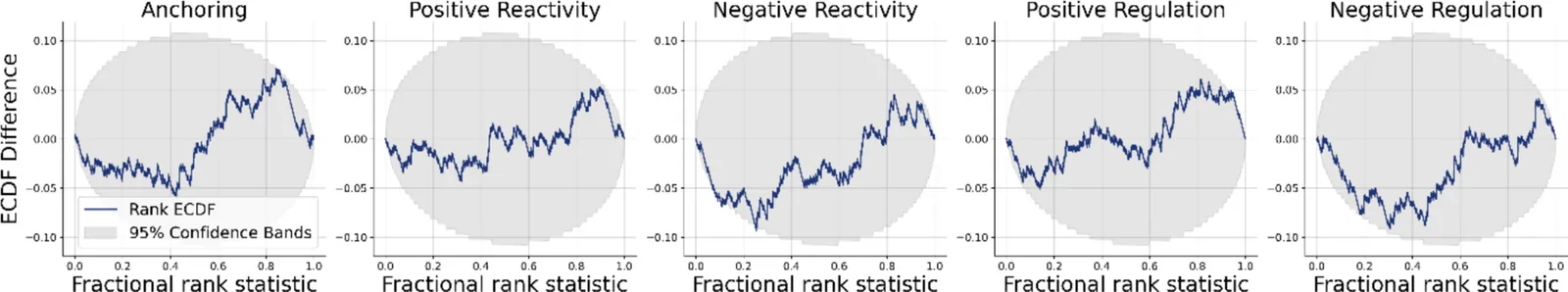
Empirical cumulative distribution function (ECDF) of rank statistics for MIVA parameters across all simulated conditions. *Note*. Simulation-based calibration check indicating no systematic biases in posterior estimation (ECDFs within confidence bands). The plots are based on 200 simulated participants and 1,000 posterior samples per participant

The empirical cumulative distribution function (ECDF) of rank statistics is an indicator of a simulation-based calibration check (SBC; Säilynoja et al., 2022) that allows for visually detecting systematic biases in the approximate posteriors. Specifically, if posteriors are well calibrated (i.e., unbiased), the rank statistics should be uniformly distributed. The ECDF difference plots were obtained by subtracting the values of the expected theoretical cumulative distribution functions from the observed ECDF values. SBC was based on 200 simulated participants. For each, a parameter vector was drawn from the prior, a time series was simulated under the present study design (13 days × 10 occasions), and 1,000 posterior samples were obtained from the trained network. The ECDF in Fig. 4 is constructed from the resulting 200 rank values per parameter. For each of 200 simulated participants, we computed the fractional rank statistic of the true parameter (the proportion of the 1,000 posterior samples falling below the true value). Under a well-calibrated posterior, these ranks are uniformly distributed across SBC iterations. Figure 4 plots the difference between the empirical and uniform cumulative distribution functions, so the expected curve is the horizontal line at zero, with the gray 95% confidence band. Deviations outside the band would indicate miscalibration: A U-shape points to over-confident posteriors, an inverted U-shape to under-confident posteriors, and a one-sided deviation indicates a directional bias. As can be seen in Fig. 4, the ECDF difference for all MIVA parameters across all conditions was within the 95% confidence bands, indicating no systematic biases in posterior estimation.

#### Calibration error

The calibration error evaluates whether posterior uncertainty correctly reflects the true uncertainty of the parameters. For each credibility level $\alpha \in \left(\mathrm{0,1}\right)$, we compute the posterior interval

3 $${I}_{\alpha ,i,k}=\left[{Q}_{\frac{1-\alpha }{2}},{Q}_{1-\frac{1-\alpha }{2}}\right],$$

where ${Q}_{q}$ denotes the q th posterior quantile. Thus, the empirical coverage of each marginal posterior is given by

4 $${\widehat{C}}_{\alpha ,k}=\frac{1}{N}\sum\nolimits_{i=1}^{N}1\left({\theta}_{i,k}\in {I}_{\alpha ,i,k}\right)$$

Calibration error summarizes the deviation between nominal and empirical coverage across credibility levels:

5 $${Calibration\,Error}_{k}={\int}_{0}^{1}\mid {\widehat{C}}_{\alpha ,k}-\alpha \mid d\alpha$$

**Fig. 5:**
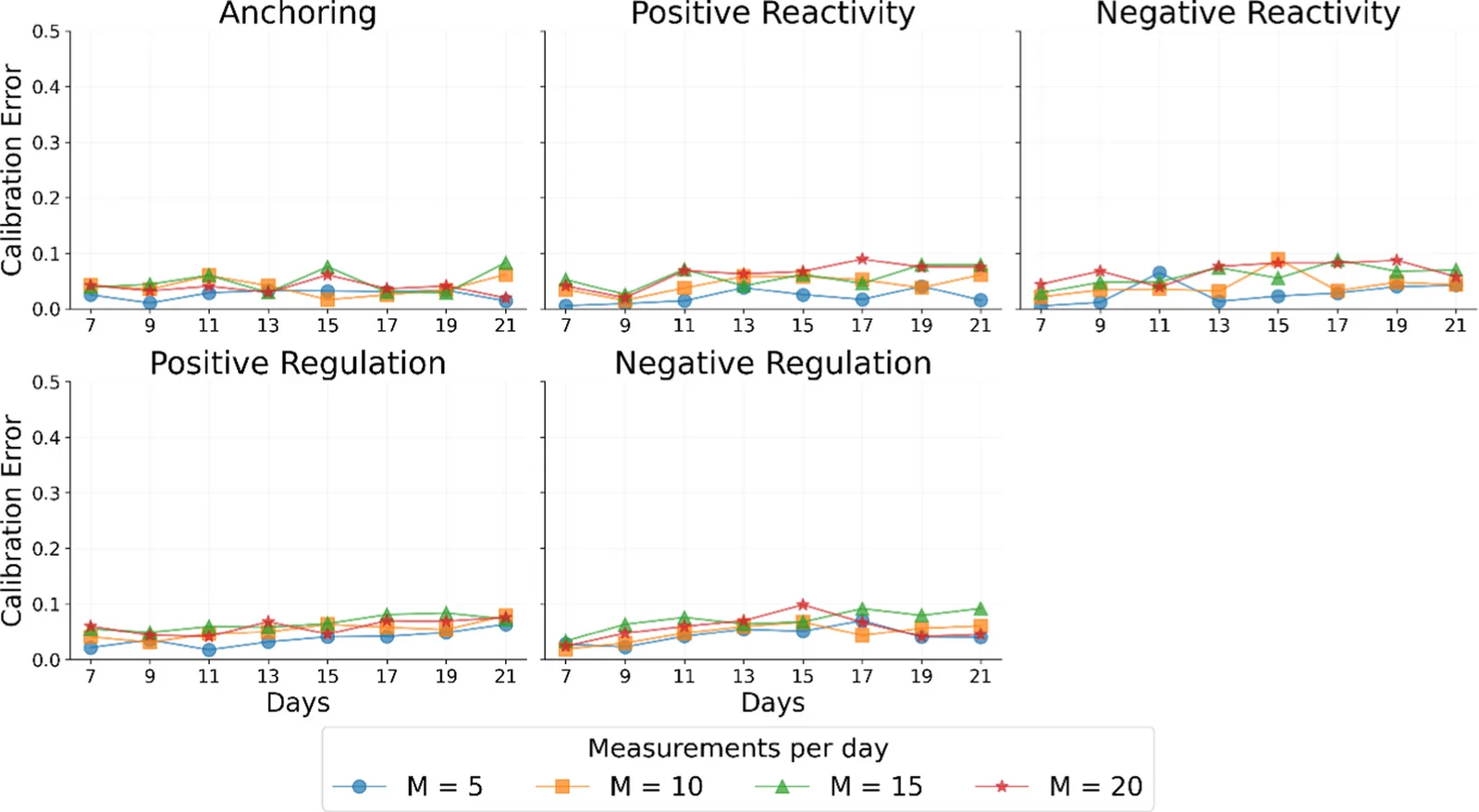
Calibration error for MIVA parameters across days (7–21) and measurements per day (5–20). *Note.* Calibration error for 500 simulated datasets and 1,000 posterior samples per condition

In practice, we approximate the integral using a finite grid of credibility levels between 0 and 1. Lower values indicate better calibrated posterior uncertainty. As can be seen in Fig. 5, there was no indication of undesirable patterns, with calibration error remaining below 10% across all study design conditions.

#### Normalized root-mean-squared error (NRMSE)

The Bayes NRMSE evaluates absolute parameter recovery accuracy while accounting for posterior uncertainty. For each posterior draw s and parameter k, the root mean squared error (RMSE) across N test datasets is

6 $${RMSE}_{k}^{\left(s\right)}=\sqrt{\frac{1}{N}\sum\nolimits_{i=1}^{N}{\left({\widehat{\theta }}_{i,k}^{\left(s\right)}-{\theta}_{i,k}\right)}^{2}}$$

To make parameters comparable across scales, the RMSE is normalized by the empirical range of the true parameters

7 $${NRMSE}_{k}^{\left(s\right)}=\frac{{RMSE}_{k}^{\left(s\right)}}{Range(k)}$$

Finally, the metric aggregates across posterior draws, with lower values indicating more accurate parameter recovery.

8 $${Bayes \,NRMSE}_{k}={median}_{s=1}^{S}\left({NRMSE}_{k}^{\left(s\right)}\right)$$

**Fig. 6:**
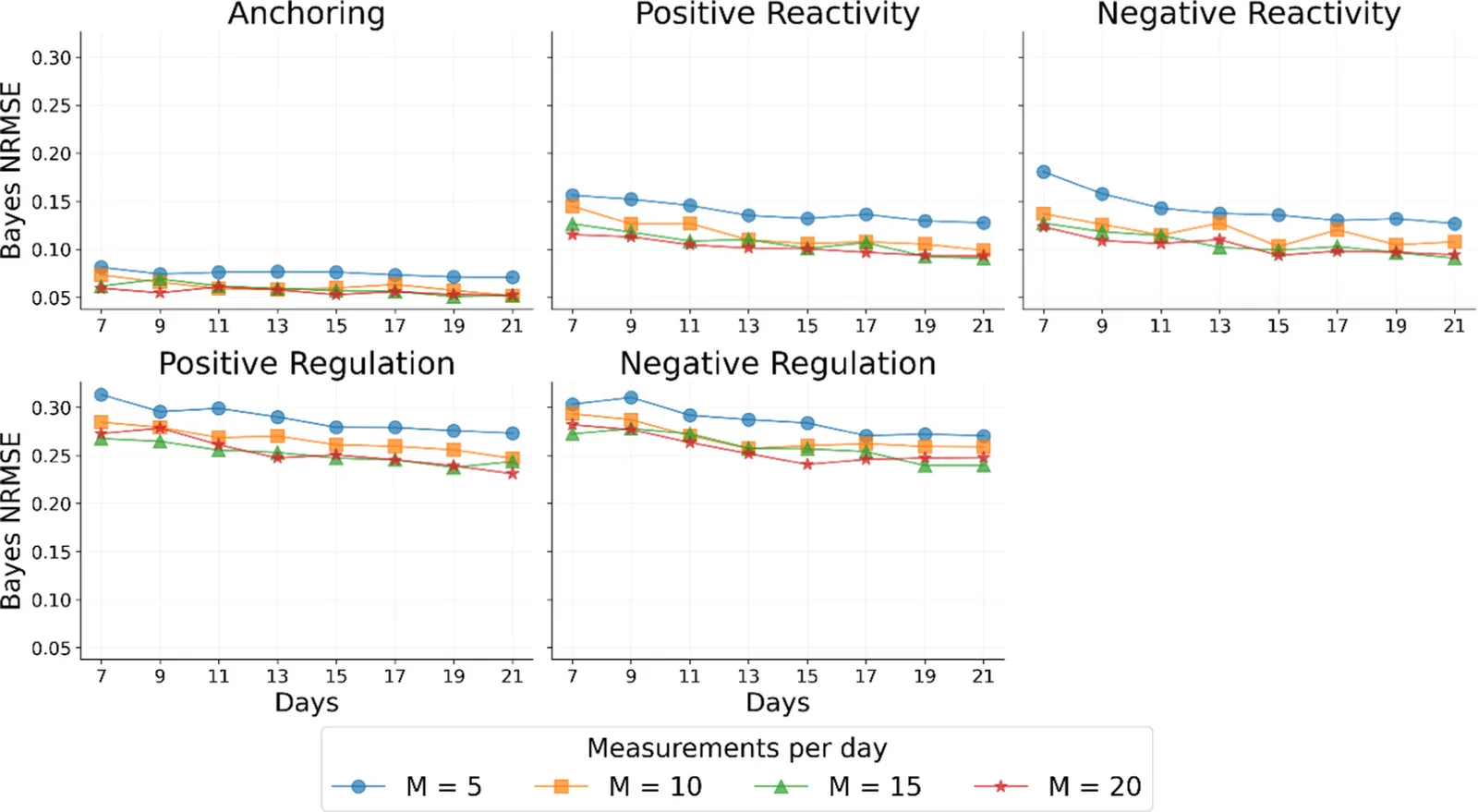
NRMSE for MIVA parameters across days (7–21) and measurements per day (5–20). *Note.* NRMSE between posterior samples and ground-truth parameters for 500 simulated datasets and 1,000 posterior samples per condition

As can be seen in Fig. 6, NRMSE decreased with increasing number of days, whereas the number of measurement occasions was less relevant for the NRMSE. Overall, parameter recovery was most accurate for anchoring, with regulation parameters showing somewhat lower accuracy.

#### Correlation

**Fig. 7:**
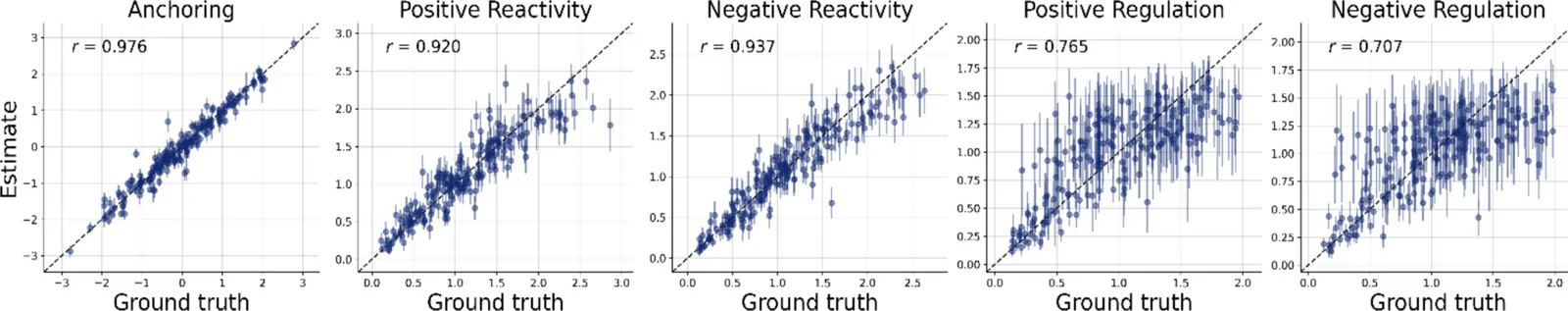
Bayes correlation for MIVA parameters for one simulated condition (13 days and 10 measurement occasions). *Note*. Points and vertical lines indicate posterior medians ± median absolute deviation, respectively

The correlation indicates the linear relation between inferred and true parameter values, with higher values (closer to 1) indicating better recovery. When looking at the correlations for one simulation condition (13 days and 10 measurement points; Fig. 7) across 200 simulated participants and 1,000 posterior samples per participant, we see that there is a good match between ground truths (true parameter values) and point estimates.

**Fig. 8:**
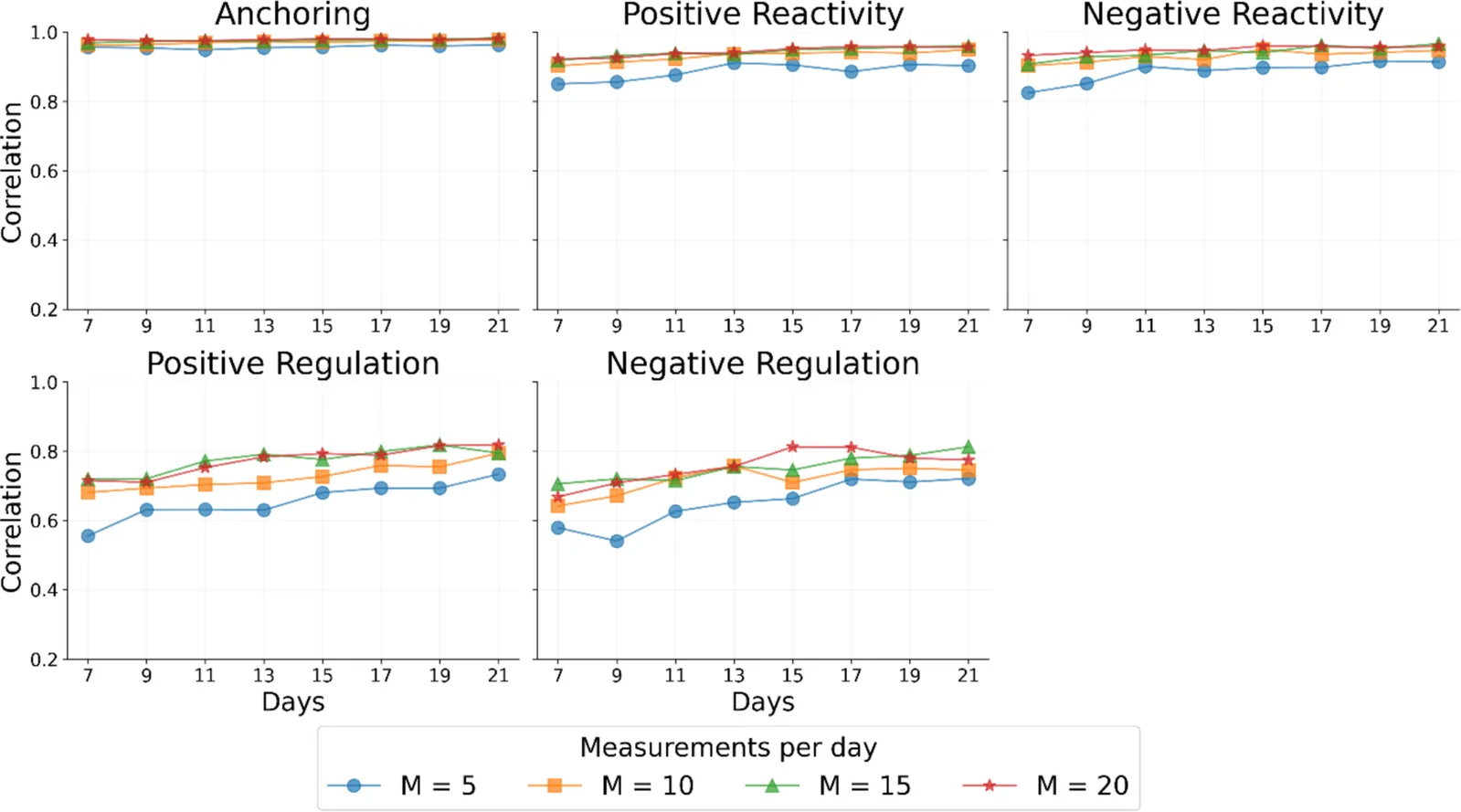
Bayes correlations for all five MIVA parameters across days (7–21) and measurements per day (5–20). *Note*. Correlations between posterior samples and ground truth parameters for 500 simulated datasets and 1,000 posterior samples per configuration

Correlations across all conditions were highest for the anchoring parameter and lowest for the two regulation parameters. Given the overall high recovery of the anchoring parameter, there were no substantial increases in correlation size if days or measurement occasions were increased. For all other MIVA parameters, increasing the number of days or the number of measurement occasions resulted in small to moderate increases in correlation size, with the regulation parameters showing the highest increase (Fig. 8). Given that an increase in either the number of days or measurements per day did not necessarily result in meaningful increases in correlations between true and recovered values, we will review the correlation results with a focus on relatively few data points.

For the reactivity parameters, correlations above .80 and below .90 were found for 28 (reactivity to positive events) and 27 (reactivity for negative events) simulation conditions, with as few as 35 measurement points (7 days and 5 occasions). Correlations equal or above .95 were found for seven (reactivity to positive events) and eight (reactivity for negative events) simulation conditions, with about 255 measurement points (17 days and 15 occasions) for reactivity to positive events and 150 measurement points (15 days and 10 occasions) for reactivity to negative events. The combination of the highest number of days (21) and measurement occasions (20) yielded high recovery (*r* = .96).

For the regulation parameters, for conditions with few days (7, 9) and few measurement occasions (5), correlations were between .54 and .63. Correlations between .70 and .80 were found for 20 (regulation of positive event impact) and 21 (regulation of negative event impact) simulation conditions, with 105 or 85 measurement points (7 days and 15 occasions for regulation of positive event impact and 17 days and five occasions for regulation of negative event impact). Correlations above .80 and below .90 were found for three simulation conditions, with 285 or 300 measurement points (19 days and 15 occasions for regulation of positive event impact and 15 days and 20 occasions for regulation of negative event impact). Correlations equal to or above .90 were not achieved with the simulated conditions. The combination of the highest number of days (21) and measurement occasions (20) yielded correlations between .77 and .82.

To summarize, we found no indication of systematic biases in posterior estimation, the posterior uncertainty reflected the true uncertainty of the parameters well, the NRMSE decreased with increasing number of days, and correlations between simulated and estimated parameters were moderate to high, depending on the MIVA parameter in question. Given that the MIVA model was assumed to be the true data-generating process, we additionally ran a simulation study with the same design parameters but in which the translation between events and affective experience was different. Specifically, it was assumed that in some cases, events had no impact on affective experience, and in other cases, events were not reported but still had an effect on affective experience. Results of this simulation study can be found at https://osf.io/spxf8/files/pr2xq. Generally, performance validation indicators were robust concerning this misspecification (for robustness of amortized Bayesian inference in light of contaminated observations, see Wu et al., 2026).

## Discussion

In this manuscript, we presented an outline of the theoretical background of MIVA. The model captures three processes assumed to be fundamental for the elicitation and fluctuation of affective experience, namely, anchoring, reactivity, and regulation (Hamaker et al., 2015; Krone et al., 2018; Kuppens et al., 2010; Larsen, 2000; Röcke & Brose, 2013). We further situated the MIVA model within the affect dynamics literature and compared it to other common computational models.

To elucidate the conditions under which it would produce reliable estimates, we conducted a simulation study that capitalized on recent advances in generative deep learning and neural network architectures (Kühmichel et al., 2026; Radev et al., 2022, 2023). Our simulation study demonstrated that the synthesis between a computational model and probabilistic neural networks results in an efficient and flexible tool for model-based inference of affect dynamics.

### Practical implications

Our simulation study showed that the anchoring and reactivity parameters can be recovered with high precision with as few as 35 data points. To achieve high precision in recovery of the regulation parameters, additional data points were needed, with at least 15 days and 10 measurement occasions. Around 150 measurement points might seem a lot, given the average ESM design with 7 days and six measurement occasions (Pirla et al., 2022; Wrzus & Neubauer, 2023). However, doubts have been raised about whether these designs provide sufficient data to adequately capture affect dynamic processes (Haslbeck & Ryan, 2022; Kuppens et al., 2022; Schuurman et al., 2015). Moreover, simulation studies for other model-based approaches also indicate that around 150 to 200 measurement points are necessary to assess affect dynamic indicators with high precision (Adolf et al., 2017; Deboeck et al., 2009; Kuppens et al., 2010; Wood et al., 2018). Other approaches used to extract indicators of regulatory processes from ESM data using lagged effects of stressful events (Scott et al., 2017; Smyth et al., 2018; Wrzus et al., 2015) might be equally disadvantaged when it comes to relatively short assessment periods.

An interesting question is why the estimation of regulation parameters requires more measurement points than the reactivity parameter. This is because the long-term effects of events on affective experience are more easily discernible from the short-term effects of subsequently occurring events (i.e., reactivity) if more data points are available. With few data points stretched out across several hours, it becomes more difficult to accurately model the unobserved affective experience. Also, with few data points, the likelihood of events being reported on successive measurement occasions increases, again leading to problems in differentiating between the short- (reactivity) and long-term (regulation) effects of events on affective experience (Almeida et al., 2020; Scott et al., 2017; Smyth et al., 2018).

Although increasing the number of days or measurement occasions both lead to similar increases in recovery of regulation and reactivity parameters, if the subject of investigation is an individual’s average affect fluctuations, the assessment period should include both weekdays and weekends as well as work and non-work times (Ram et al., 2014; Scott et al., 2020). When interested in affect dynamics related to a specific event (e.g., university exam; Dejonckheere et al., 2021), increasing the number of assessments per day before and after this event might be recommended, as there are more within-person affect fluctuations within than across days (Scott et al., 2020).

Generally, assessing affect dynamics at time scales that do not map onto those at which the underlying processes unfold may result in misleading or inaccurate inferences (Hamaker, 2025; Haslbeck & Ryan, 2022; Lazarus et al., 2021). Very frequent measurements with short time intervals can lead to a proportional increase in measurement error compared with true change (Boker & Nesselroade, 2002; Hopwood et al., 2022). Too sparse measurement could lead to missing important micro changes in affect (Hamaker & Wichers, 2017; Kalokerinos et al., 2020; Kuppens et al., 2022). Most importantly, the appropriate temporal resolution of measurements should be informed by theoretical work on affective change. However, only a few theoretical ideas concerning the time scale on which affective changes occur in the real world have been put forward (Fried, 2020; Hopwood et al., 2022; Lazarus et al., 2021). The Flex3 model (Hollenstein, 2015, 2021) proposes that real-time, temporally adjacent shifts from one affective state to another capture affect regulation. Other affect dynamic concepts assume a less intricate temporal resolution and propose cyclic changes in affective experience across weeks, months, or seasons (Chow et al., 2005; Larsen & Kasimatis, 1990; Liu & West, 2016; Ollero et al., 2023). However, current model-based approaches to affect dynamics, including MIVA, have not formulated specific assumptions about the exact time-dependent nature of changes in affective experience. Rather, the timing of affect fluctuations is implicitly assumed to happen on a minute-to-hourly basis. The specific focus of MIVA was to understand how events elicit affective fluctuations and how much and how long they influence the underlying affect processes. This focus was inspired by work calling attention to the neglected role of event appraisals in affect dynamic research (Vanhasbroeck et al., 2022; Wenzel et al., 2022). Going beyond this event-based focus and integrating an in-depth exploration of time-related processes will greatly enhance our understanding of affect dynamics.

### Strength and limitations

To complement current methods and models of affect dynamics, we developed a theory-based approach that specifically focuses on the effects of events on the processes assumed to underlie affect dynamics. The MIVA parameters anchoring, reactivity, and regulation capture affect characteristics that differ between individuals, are relatively stable across time, and are relatively consistent over situations. Its parameters reflect essential processes that account for observed changes in affective experience (Wirth et al., 2022).

#### Event dependence

While focusing on the short- and long-term effects of events affords a closer look at how situational appraisals are translated into affective fluctuations, it comes at the expense of having to rely on participants reporting such events. The MIVA parameters regulation and reactivity are conceptualized as event-dependent, meaning that they cannot be estimated for participants who do not report any events. Thus, in our simulation study, we also had to constrain the used datasets to those containing at least two events. The event-based nature of MIVA limits its applicability for empirical datasets of everyday affective experience. Research has just started exploring the characteristics of samples in ESM studies that do not report events in daily life (Charles et al., 2021). How well their affect dynamics can be investigated under controlled laboratory conditions remains an open question. Adopting MIVA for datasets from controlled laboratory studies is an important future avenue because it allows us to (a) determine affect dynamics of individuals who do not experience or report daily events, (b) examine affect dynamics with a high temporal resolution on a second-to-second basis, and (c) shed light on the yet unresolved issue of overlap between affect dynamics assessed in laboratory versus daily life (Davidson, 2015; Klein et al., 2023; Koval et al., 2015; Kuppens et al., 2022).

#### Cyclic changes and changes in model parameters over time

To keep MIVA as parsimonious as possible, it captures only certain parts of the complex reality. It currently does not include parameters for cyclic changes in affective experience. Such intricacies have not been incorporated because the model then may become harder to manage both conceptually and computationally, requiring large sample sizes and more measurement occasions to estimate the model parameters with reasonable precision (Bosse et al., 2014; Krone et al., 2018). Additionally, MIVA currently rests on the assumption that its parameters are stable over time. However, due to critical life events, developmental processes, or interventions, the model parameters may exhibit changes that are more or less transient (Bringmann et al., 2016; Koval & Kuppens, 2012). Statistical advances have accounted for changes in affect dynamic indicators, such as the continuous time dynamic modeling approach (Driver & Voelkle, 2018) or the time-varying change point autoregressive model (Albers & Bringmann, 2020). These statistical approaches, however, are descriptive and exploratory rather than driven by specific theoretical ideas of time-dependent changes in affect dynamics. What is needed is a deeper understanding of the time course at which affect fluctuates and how this time-dependent change maps onto the frequency with which studies investigate affect dynamics. This will help to develop a more comprehensive theory of affect dynamics that explains the underlying causal mechanisms and makes accurate predictions about the magnitude, shape, and direction of affect fluctuation and the time scales and contextual conditions under which they occur (Fried, 2020; Lazarus et al., 2021). Otherwise, the field is left with an abundance of exploratory and pluralistic approaches and difficulties in determining which indicators are theoretically meaningful (Kuppens et al., 2022).

#### Range of applicability of the model

Our simulation study demonstrated the utility of the amortized Bayesian inference as implemented in the BayesFlow toolkit for assessing affect dynamics. We have shown that the same trained network can be utilized across different study designs. This makes the method especially effective when inferences are drawn across different scenarios with a large number of datasets and/or varying numbers of participants or assessment occasions. The reuse of trained networks for multiple datasets and across multiple researchers will increase the comparability and reproducibility of findings in affect dynamic research. Going beyond the current simulation study, the posteriors estimated via the BayesFlow method can be used to test hypotheses about particular parameter values, compute individual differences, or compare means between conditions in a fully Bayesian way. When focusing especially on the comparison of affect dynamics between different groups (e.g., age groups or patient groups), using hierarchical BayesFlow models including hyperpriors would be advisable. To increase the applicability and usability of the novel simulation-based BayesFlow method, developing and maintaining user-friendly software is crucial. So far, BayesFlow offers a user-friendly application programming interface (API) which encapsulates the details of neural network architectures and training procedures. Concerning MIVA, our simulator, approximation scripts, and results are fully open and reproducible. Thus, we hope that amortized Bayesian inference will enhance model-based inference in affect dynamic research.

## Conclusion

Affective experience is inherently dynamic, as it continuously fluctuates as a result of internal or external events. We applied a formalized theoretical approach to develop a parsimonious model of affect dynamics. MIVA allows estimating parameters for anchoring, reactivity, and regulation based on affective states in combination with daily events. MIVA was empirically evaluated by conducting a simulation study using amortized Bayesian inference, a novel approach capitalizing on the latest developments in machine learning. Our simulation demonstrates the applicability of amortized methods for studying affect dynamics and offers insight into the conditions under which the MIVA model parameters can be estimated with high precision.

## Data Availability

Data included in this manuscript are available at https://osf.io/spxf8/.

## Footnotes

1 Affective fluctuations are also studied in affective computing research. However, affective computing usually focuses on developing artificial systems and devices that can recognize, interpret, process, and simulate human affect (Bosse et al., 2014; Ryan et al., 2025).

2 However, this is not necessarily true for event ratings. If event ratings are assessed on two unipolar scales with values > 0, the impact of unpleasant events can lead to a decrease in affect instead of adding the product of ${Ru}_{i} \bullet { Iu}_{ij}$; this product is subtracted such that

$${A}_{ij}=\frac{r}{1+ {e}^{-{(a}_{i}-{ Ru}_{i}\, \bullet \,{ Iu}_{ij}+{ Rp}_{i}\, \bullet \,{ Ip}_{ij})}}- \frac{r}{2}$$

3 Dependence on scale range can be avoided by standardizing. Centering the affect scale around zero is not strictly necessary for model estimation, but it provides meaningful advantages for interpretation. When affect is centered, the anchoring parameter directly reflects deviations from a neutral affective state: a = 0 indicates neutral baseline affect, positive values indicate a positive affective disposition, and negative values indicate a negative disposition. Additionally, centering ensures that the mathematical formulation of the decay functions naturally returns affect toward a psychologically meaningful neutral point rather than toward an arbitrary numerical value.

4 In principle, it would be possible to extend the MIVA model to include multidimensional affect measures. Different reactivity and regulation parameters could be estimated for each dimension of affective experience, and correlated errors could be used to bind these processes together. As the numbers of parameters would substantially increase in such a model, probably more data is required to reliably identify the interrelations of affect dimensions.

5 To compare the speed of parameter estimation, MIVA parameters were estimated from 20 simulated datasets with 14 days of observations and 20 measurements per day using a Stan model. On a modern CPU (AMD Ryzen 7 at 3.80 GHz), fitting the model with 2,000 MCMC samples per chain (four chains run in parallel) took about 4 × 24 s per dataset. Parameter estimation for 1,000 data sets, 8 × 4 different combinations of days and measurements per day, four chains and assuming 24 s for each chain; would take approximately 35.5 days.

6 While the forward model is an explicit, sometimes complex simulator $(p\left(x\right|\theta ))$ that generates data from parameters, the inverse mapping $p\left(\theta \right| x)$ is generally intractable. Amortized SBI trains a conditional density estimator to directly approximate this inverse mapping.

7 This is done to create a static, deterministic model that can be used for inference. Parameters are locked, and no further learning (updating of weights via backpropagation) occurs when processing new data.

8 While determining the optimal number of events for good or excellent recovery of MIVA reactivity and regulation parameters is beyond the scope of this manuscript, initial exploration has shown that on average, few (< 1 event per day) and too many events (> 3 events per day) may impede recovery.

## Code availability

Scripts included in this manuscript are available at https://osf.io/spxf8/.
